# Characterization of the interaction of staphylococcal enterotoxin B with CD1d expressed in human renal proximal tubule epithelial cells

**DOI:** 10.1186/s12866-015-0344-5

**Published:** 2015-02-04

**Authors:** Rasha Hammamieh, Nabarun Chakraborty, Yixin Lin, Jeffrey W Shupp, Stacy-Ann Miller, Sam Morris, Marti Jett

**Affiliations:** Integrative Systems Biology, US Army Center for Environmental Health Research Fort Detrick, 568 Doughten Drive, Fort Detrick, MD 21702-5010 USA; Axela, Inc., 50 Ronason Drive, Suite 105, Toronto, ON M9W 1B3 Canada; The Burn Center, Department of Surgery, Washington Hospital Center, Washington, DC 20010 USA

## Abstract

**Background:**

Participation of renal cells in the pathogenesis of staphylococcal enterotoxin B (SEB) is critical for late cleansing and sequestration of the antigens facilitated by CD1d mediated antigen sensing and recognition. This is a noted deviation from the typical antigen recognition process that recruits the major histocompatibility complex class II (MHC II) molecules. The immunological importance of CD1d is underscored by its influences on the performances of natural killer T-cells and thereby mediates the innate and adaptive immune systems.

**Results:**

Using diffraction-based dotReady™ immunoassays, the present study showed that SEB directly and specifically conjugated to CD1d. The specificity of the conjugation between SEB and CD1d expressed on human renal proximal tubule epithelial cells (RPTEC) was further established by selective inhibition of CD1d prior to its exposure to SEB. We found that SEB induced the expression of CD1d on the cell surface prompting a rapid conjugation between them. The mRNA transcripts encoding CD1d remained elevated potentially after completing the antigen cleansing process.

**Conclusion:**

Molecular targets associated with the delayed pathogenic response have essential therapeutic values. Particularly in the event of bioterrorism, the caregivers are typically able to intervene much later than the toxic exposures. Given circumstances mandate a paradigm shift from the conventional therapeutic strategy that counts on targeting the host markers responding to the early assault of pathogens. We demonstrated the role of CD1d in the late stage of pathogen recognition and cleansing, and thereby underscored its clinical potential in treating bioweaponizable antigens, such as Staphylococcal enterotoxin B (SEB).

**Electronic supplementary material:**

The online version of this article (doi:10.1186/s12866-015-0344-5) contains supplementary material, which is available to authorized users.

## Background

SEB, a member of the exotoxin family produced by staphylococci [1,2], is a superantigen (sAg) capable of inducing toxic shock through intranasal or intravenous portals. Transmittable via air, food and water, the SEB-induced toxemia causes pyogenic damage that manifests as immunological irregularities, arthritis, and autoimmune disease cascading to multi-organ dysfunction and lethal consequences [1,3]. Continued clinical interest about staphylococcal enterotoxins (SEs) is attributed to a general inadequacy of its effective treatment [3,4]. Many incidences alleging SEB as the chief food poisoning contaminant [5] and its easy adaptability to bioweapons [6] further justify its continued clinical relevance.

Administrations of the anti-toxin and anti-inflammatory agents are the typical clinical strategies available nowadays [3,7]. Conventional strategies targeting early pathogenic markers have faced some serious deficiencies particularly when they intervene long after the pathogenic assault [8]. This concern has been multiplied by the failure of the conventional therapeutic strategy in treating septic shock [9], which shares many patho-clinical similarities with toxic shock [10,11]. In the advent of chemical warfare, therapeutic interventions typically lag behind the toxic assault. Hence, the molecular signatures involved with the delayed response to bioweaponizable toxins, such as SEB, are of significant clinical interest.

Accumulating evidences suggest robust clinical efficacy in targeting the post-assault downstream candidates responsible for SEB pathogenesis [3,12]. In recent past, the focus of investigation for clinical targets has shifted from blood cells to the non-lymphoid cell types derived from kidney [13], spleen [14], lungs [15], and gut [16]. Recent findings demonstrating the translocation of bacterial infection from skin [17] to organs, such as kidney and spleen without hematic invasion, further enhanced the interests on such peripheral organs in this context. It was also noted that many cell types [18,19] express atypical binding sites for SEB, which underscore the essentiality of cell/tissue-specific studies. In other words, CD1d (cluster of differentiation 1d glycoprotein) expressed on kidney cell surface may demonstrate a very unique response characteristics to SEB.

The critical role of the kidneys in exotoxin clearance and sequestration [20-25] was attested by identifying the renal excretion process as the primary route of removing staphylococcal enterotoxin A (SEA) from plasma [26]. Supporting evidence includes the non-human primate study reporting ~70% accumulation of SEB in the renal proximal tubule epithelial cells (RPTECs) within 90 minutes of aerosol administration [22]. SEB introduced through a cutaneous burn wound of rat was found traversed to and localized in the kidney 6 days post-burn [27], and a murine genomic study showed a delayed response of renal genes after lethal SEB shock [12]. Further investigation of the renal response in SEB pathogenesis may help to identify an alternative avenue for clinical intervention.

The present study is focused on CD1d for two primary reasons. Firstly, CD1d selectively expressed in the renal epithelial tissues are recruited for antigen sensing, recognition and cleansing at the late stage of pathogenesis [13,28-31]. Unlike a typical host-mediated antigen recognition sequence, the intact sAgs are submitted to the antigen presenting cells (APC) by CD1d, a phylogenetic analog of major histocompatibility complex (MHC) class I and II molecules operating as an antigen-trafficking agent [32-34]. Secondly, CD1d controls the function of natural killer T-cells (NKT cells); the CD1d-NKT cell-mediated sAg recognition event rapidly triggers the release of many cytokines, and thereby systematically influences the hosts’ adaptive and innate immune systems [35,36].

The involvement of CD1d in pathogenesis has been investigated in the past, primarily focused on the lymphoid cells [37-41], while the knowledge gap exists in comprehending the CD1d-mediated pathogenesis in kidney cells and its role in the late stage of pathogenesis. To bridge the knowledge gap, our study sought to understand the renal response to SEB shock, focusing on the interaction of SEB with CD1d in RPTECs.

## Methods

### Cells and reagents

Human RPTECs were purchased from Lonza (Frederick, MD). SEB and biotinylated SEB (bt-SEB) (95% pure, vendor defined) were purchased from Toxin Technology, Inc. (Sarasota, FL). CD1d-(Immunoglobin) Ig recombinant protein, which has extracellular MHC class I-like domains of the human CD1d molecule fused with the V_H_ regions of mouse IgG1, was purchased from BD Biosciences (San Jose, CA). Horse radish peroxidase (HRP)-linked anti-mouse (H + L) horse antibody (HRP-anti-mouse) was obtained from Cell Signaling Technology, Inc. (Danvers, MA). Mouse IgG (msIgG), a polyclonal antibody purified from pooled mouse serum by fractionation and ion-exchange chromatography, was from Axela, Inc. (Toronto, Ontario, Canada). Biotin-SP-AffiniPure Donkey Anti-Rabbit IgG (H + L) antibody (bt-Dar) was from Jackson ImmunoResearch Laboratories (West Grove, PA). 1-Component TMB (3,3',5,5'-Tetramethylbenzidine) Membrane Peroxidase Substrate (a precipitable form of TMB) was from KPL, Inc. (Gaithersburg, MD). All other antibodies used in this study were purchased from Abcam, Inc., Cambridge, MA.

### Diffraction-based dotReady™ immunoassay

The assays were performed in biosensors with avidin surface chemistry (Axela, Inc.). All experiments were carried out with the dotLab® System (Axela, Inc.).

#### Principle of dot®

The dot® (diffraction optics technology) utilizes diffraction grating to analyze real-time protein-protein interactions. A capture reagent is immobilized on a specific pattern of lines on the surface of a prism-shaped dotLab® Sensor. The sensor surface forms the base of a 10 μL flow cell. A series of discrete diffraction beams are generated when illuminating the underside of each assay spot with a laser. The subsequent capture of the binding partner alone or bound to a detector antibody increases the average height of the surface pattern and causes an increase in the diffraction intensity (DI) signal that is recorded in real-time. The DI signal is directly related to the size and quantity of the bound complex [42,43]. The size of the bound complex is not limited to the addition of proteins, as previous studies [44] have shown that oxidization of TMB, a precipitable form of HRP substrate, can cause specific and localized precipitations on the sensor surface and result in a significant increase of DI signals.

The principle mechanism governing the assay performance is very similar to the other industry standards such as the BIAcore with a primary difference in their tethered ligand characters [45]. Many studies reported comparable performance of dot® technology in comparing other platforms such as BIAcore, amperometric assay or other methods for detecting real-time bimolecular interactions [46-48].

#### dotReady® assay

**Figure 1:**
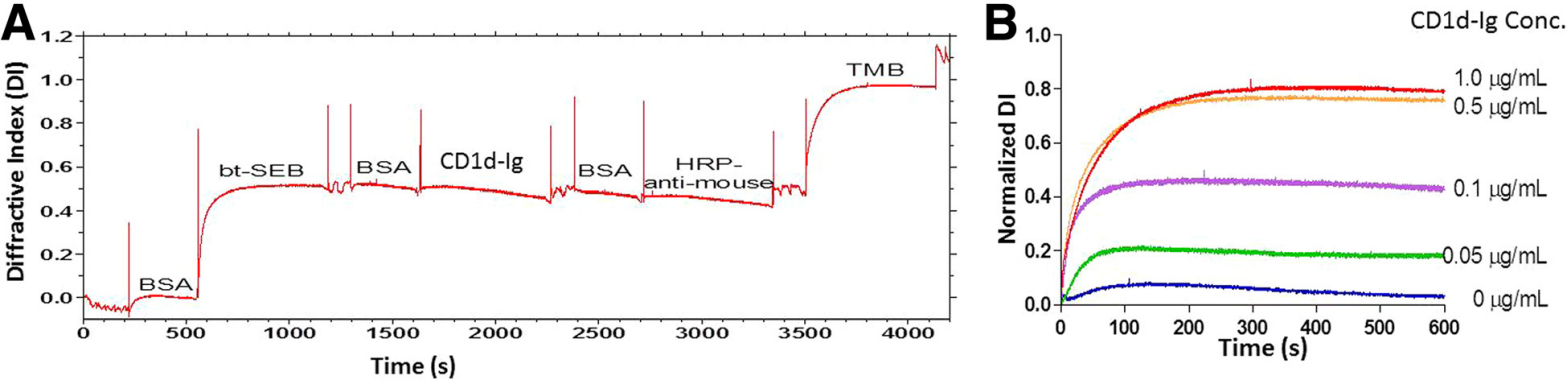
**The kinetics and specificity of the capture of CD1d with biotinylated SEB (bt-SEB) with the dotReady system were characterized. A.** Kinetics of SEB-CD1d interaction: A representative real-time trace depicts the kinetics of capturing CD1d with biotinylated SEB (bt-SEB) on the dotReady™ system. Here, the X-axis indicates the assay duration in seconds (s) and the Y-axis indicates measured diffractive index (DI). 10 μg/ml bt-SEB was immobilized on the avidin sensor during a 10 m incubation. CD1d:Ig fusion protein (extracellular major histocompatibility complex (MHC) class I-like domains of human CD1d fused with V_H_ regions of mouse IgG1) (1 μg/ml) was added and incubated for a 10 m post-BSA treatment. After a second round of BSA washing, the complex was incubated with horse anti-mouse HRP-conjugated secondary antibody (HRP-anti-mouse; 1 μg/ml) for another 10 m. Finally, TMB was added as the reporting agent. The shift of DI with time in seconds is presented herein. For all DI tracing, the upward spikes are air gaps separating reagents. All non-labeled portions are attributed to the wash steps. Increased DI signals showing as an upward ramp indicated the binding of a reagent in this step. The highest ramp after TMB presentation suggests the successful binding of SEB and CD1d. **B.** Signal increases with increasing concentrations of CD1d presented to bind SEB: The assay followed the same sequential addition described in Figure 1A, namely, the presentation of biotinylated SEB to avidin sensor, followed by addition of CD1d:Ig fusion protein, horse anti-mouse HRP-conjugated secondary protein and TMB, respectively intercepted with BSA washing. The normalized DI signals measured during 600 s after the introduction of TMB were plotted against the serially increasing concentrations of CD1d from 0 to 1.0 μg/ml. A sigmoidal dose–response (variable slope) function was used to fit the curve, R^2^ = 0.9984. The positive correlation of the increased concentration of CD1d:Ig with enhanced DI while keeping the concentration of the other reagents constant validated the assay specificity.

An avidin sensor was washed with running buffer containing PBS (0.154M NaCl, 0.01M phosphate, pH7.4) with 0.025% (v/v) Tween-20, and the surface was blocked to minimize nonspecific binding using bovine serum albumin (BSA) blocking buffer (5 mg/ml of BSA in running buffer) for 5 m in a mixing mode that repeatedly reverses the flow directions within the sensor. This mode was used in all subsequent incubations unless noted otherwise. Subsequently, the sensor was incubated with 10 μg/ml of bt-SEB for 10 m and washed with running buffer. BSA blocking buffer was applied and incubated for 5 m. The dimer of CD1d:Ig at 1 μg/ml was added and incubated for the next 10 m. The sensor was washed with running buffer prior to a 5 m incubation of the BSA blocking buffer. HRP-anti-mouse horse antibody at a concentration of 1 μg/ml was added to the sensor and incubated for 10 m. The sensor was washed with running buffer and PBS buffer. Finally, TMB was introduced and incubated for 10 m in static mode (i.e., the flow stopped) and DI was reported (Figure 1A).

The change in DI was recorded during each loading episode. Prior to the introduction of each reagent, the sensor was blocked with the BSA blocking buffer to establish the baseline. The delta (Δ) (the change of DI signals from the introduction of one reagent to the next one) indicated the extent of binding during each step. Each binding event was independent from each other and evaluated separately. The DI signals derived from each step of sequential addition were normalized against the delta of bt-SEB binding (as “normalized DI”), thus minimizing inter-sensor variations. Such ratio comparison is typical and has been reported previously [48,49].

The specificity of CD1d in binding SEB was evaluated by systematically replacing bt-SEB with bt-Dar (Additional file 1: Figure S1A) and CD1d:Ig with BSA (Additional file 2: Figure S1B), keeping the rest of the assay protocol unchanged. Furthermore, the efficacy of CD1d:Ig fusion protein was validated with two separate experiments. In one of them, the Ig tag uncoupled from CD1d was presented instead of CD1d:Ig fusion protein, keeping the rest of the protocol unaltered. In the second experiment, HRP was presented coupled with nonspecific mouse antibody, instead of the CD1d-specific mouse antibody. The normalized DIs reported from each of the scenarios were presented along with the DI derived from the standard assay protocol described above (Additional file 3: Figure S1C)

The assay was repeated with a range of concentrations of CD1d:Ig fusion protein (0, 0.05, 0.1, 0.5, and 1.0 μg/ml), keeping the rest of the assay conditions unchanged, and the corresponding DIs for each CD1d:Ig concentration were reported in Figure 1B. All data recorded in dotLab® Software were analyzed using GraphPad Prism® (GraphPad Software, Inc., San Diego, CA).

### RPTEC culture and fluorescence-based reporting

**Figure 2:**
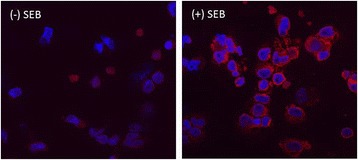
**Influence of SEB on CD1d cell surface expression: Fluorescence-based CD1d reporting: 1 x 10** ^**6**^ **cells/ml RPTECs were exposed to 100 μg/ml SEB for 1 h, washed and then probed using the mouse anti-CD1d antibody coupled to Alexa 594-conjugated goat anti-mouse (Red spots).** This image is designated as (+) SEB. The (−) SEB image was obtained from the cells treated with the same method; however, SEB was replaced with PBS. All images are at 60X magnification captured with a fluorescent microscope equipped with a digital camera (Olympus Optical Company). The red spots represent the CD1d expressed on the cell surface.

RPTECs were grown in REBM culture medium supplemented with a bullet kit containing hEGF, hydrocortisone, epinephrine, insulin, triiodothyronine, transferrin and GA-1000 (Lonza, MD). Cells were cultured and passaged as per supplier’s protocol using recommended reagents. Cells at passage 5–6 were plated in chamber slides at 1×10^6^ cells/ml for 24 h at 37°C. SEB was added to the cells at a concentration of 100 μg/ml and incubated at 37°C for 15 m, 30 m, 1 h and 2 h. Post-incubation, the cells were washed twice with 1× PBS. For the CD1d reporting assays (Figure 2), the cells were treated with mouse anti-CD1d antibody, incubated for 30 m on ice and washed with 1× PBS. Subsequently, 5 μl of Alexa 594-conjugated goat anti-mouse antibodies in 1× PBS was added and incubated for 5 m on ice and washed. The cells were resuspended in 1 ml of formaldehyde and 1× PBS mix (1:1) for 15 m at room temperature, washed twice using 1× PBS, subsequently resuspended in 1 ml of 1× PBS, and imaged using a fluorescent microscope equipped with a digital camera (Olympus Optical Company, Melville, NY). RPTECs without SEB exposure were processed similarly and imaged, and Figure 2 reports surface expression of CD1d after 1 h exposure to SEB or PBS treatment (negative control).

For the SEB reporting assays (data not shown), the cells were treated with rabbit anti-SEB antibody for 30 m on ice, washed with 1× PBS, and incubated on ice with FITC-conjugated goat anti-rabbit antibodies for 5 m. Subsequently, the cells were washed and resuspended in 1 ml of formaldehyde and PBS mix (1:1). After incubation at room temperature for 15 m, it was washed with 1× PBS, resuspended again in 1× PBS and proceeded to imaging. The images were analyzed using a fluorescent microscope equipped with a digital camera (Olympus Optical Company). The control chamber slide had no SEB loading.

**Figure 3:**
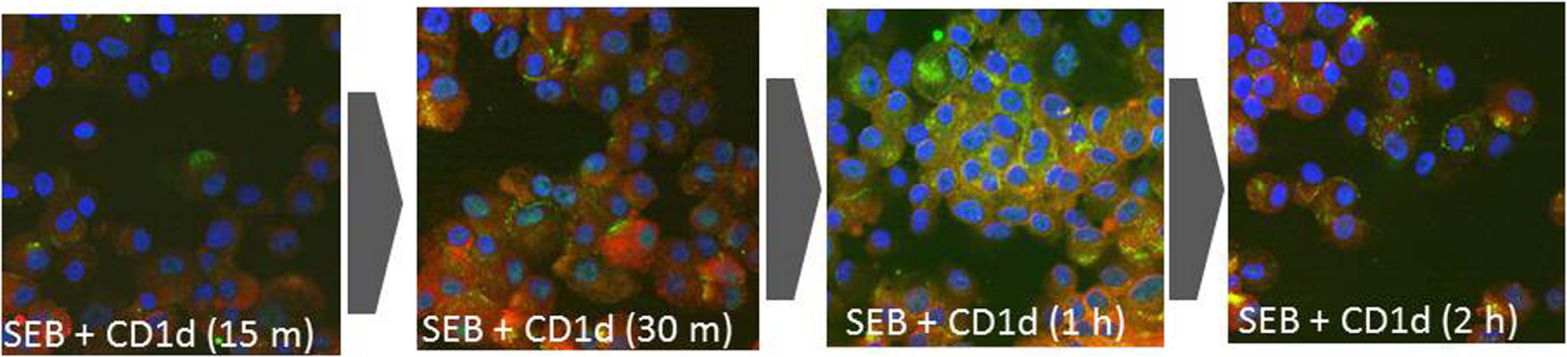
**Time course study of SEB binding to RPTECs: Fluorescence-based co-reporting of SEB and CD1d: 1 x 10** ^**6**^ **cells/ml RPTECs were exposed to 100 μg/ml SEB, washed and then CD1d and SEB were sequentially reported.** To report CD1d, mouse anti-CD1d antibodies coupled to Alexa 594-conjugated goat anti-mouse antibodies were used (Red spots). To report SEB, rabbit anti-SEB antibodies coupled with FITC-conjugated goat anti-rabbit antibodies were used (Green spots). Control: The “no SEB” treatment was similar to Figure 2 (not shown herein); SEB (15 m): SEB assault for 15 minutes; SEB (30 m): SEB assault for 30 minutes; SEB (1 h): SEB assault for one hour; and SEB (2 h): SEB assault for two hours. All images are at 60X magnification imaged with a fluorescent microscope equipped with a digital camera (Olympus Optical Company).

For dual reporting, RPTECs were harvested, plated in chamber slides and treated with SEB for increasing durations (15 m, 30 m, 1 h and 2 h), as per the protocol described earlier. Post-treatment, the cells were incubated on ice with mouse anti-CD1d antibody; after 30 m incubation, rabbit anti-SEB antibody suspended in PBS was added and the incubation on ice was continued for another 30 m. The cells were washed twice, and the Alexa Fluor 594-conjugated goat anti-mouse antibody and FITC-conjugated goat anti-rabbit antibody suspended in 1x PBS were added to incubate for 5 m. Subsequently, the cells were washed, resuspended in 1 ml of formaldehyde and PBS mix (1:1). After incubating at room temperature for 15 m, it was washed with 1× PBS, resuspended again in 1× PBS and proceeded to imaging. The images were analyzed using a fluorescent microscope equipped with a digital camera (Olympus Optical Company). Figure 3 shows the fluorescence images of 15 m, 30 m, 1 h and 2 h of SEB treatments.

### Immunoblotting to report time dependent binding efficiency of SEB to CD1d

Co-immunoprecipitation of CD1d-bound SEB was carried out using the Universal Magnetic Co-IP kit (Active-Motif, Carlsbad, CA) according to the manufacturer’s instructions. Briefly, RPTECs were exposed to SEB for 1 h and 2 h as per the protocol described earlier. An aliquot of 400 ng of whole cell extract and 2 μg of mouse anti-CD1d antibody was combined in a pre-chilled 1.5 ml tube and incubated for 2 h at 4°C on a rolling shaker. Samples were centrifuged for 30 s at 4000 rpm at 4°C, then 25 μl of Protein G Magnetic Beads was added to each tube. The mixture was incubated for 1 h at 4°C and centrifuged for 30 s at 4000 rpm at 4°C. Each tube was placed on a magnetic stand to pellet the beads, supernatants were discarded, and the pellets were resuspended and subsequently washed with 1× PBS. Immunoblotting was performed according to the protocol described elsewhere (Additional file 4: Figure S2) [50].

### qPCR assay to assess CD1d expression

The protocol described earlier was followed to harvest RPTECs and to expose them to 100 μg/ml SEB for 1 h, 2 h and 24 h. Total RNA was isolated from RPTECs in a 125 ml flask using TRIzol reagent (Invitrogen, Carlsbad, CA) as per manufacturer’s protocol. RNA was quantified via spectrophotometry followed by analysis with a Bioanalyzer 2100 (Agilent Technologies, Santa Clara, CA).

**Figure 4:**
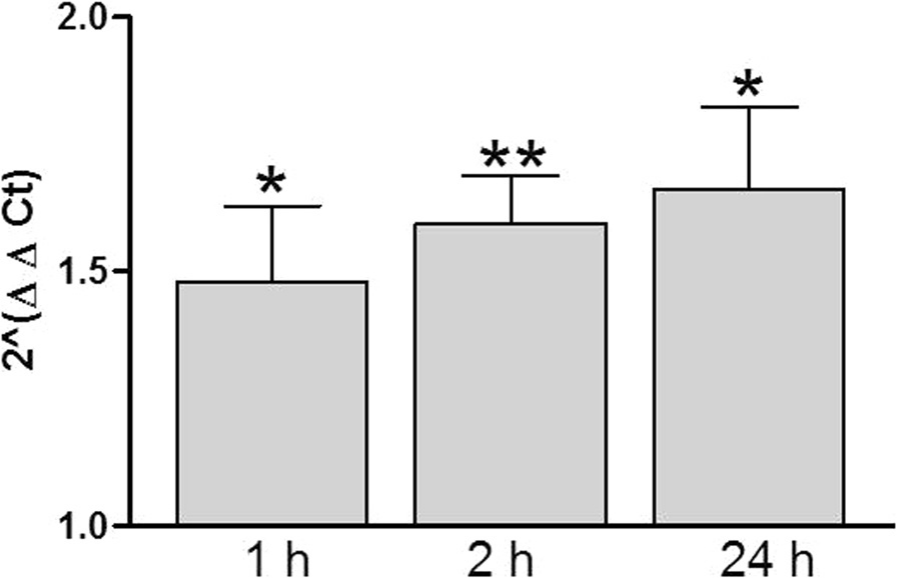
**Shift of CD1d mRNA expression with increased duration of SEB exposure: Real-time PCR was carried out on RNA extracted and converted to cDNA from SEB-exposed RPTECs.** 1 x 10^6^ cells/ml RPTECs were exposed to 100 μg/ml SEB for 1 h, 2 h and 24 h (*N* =3). Data are expressed as fold-change compared to the controls for each time point and represent the 2^ΔΔCt^ (± SE). Welch’s *t*-test *p* value cut-offs were designated by * < 0.05 and ** < 0.01.

The primer sequences used for the amplification of CD1d were 5'-GGGCACTCAGCCAGGGGACATCCTGCCCAA-3' as forward and 5'-GATACAAGTTTGCACACCTTTGCACTTCTG-3' as reverse. The specificity of each primer sequence was further confirmed by running a BLAST search. Reverse transcription and real-time PCR reactions were carried out using iScript cDNA Synthesis Kit (Bio-Rad, Hercules, CA) and Real-time PCR kit (Roche, Indianapolis, IN), respectively. Five technical replicates of each reaction were completed in an I-Cycler machine (Bio-Rad). Each sample was also amplified using a primer pair targeting 18S ribosomal RNA as the housekeeping gene, whose selection for the present purpose was instituted because of many past observations [51-53]. The resultant cycle threshold data from each real-time PCR experiment was converted to fold-change by using an established algorithm [54]. The fold-change results obtained at three time points were compared by Welch’s corrected *t*-test *p* values * < 0.05 (Figure 4).

### Immunological inhibition of CD1d fluorescence-based reporting

**Figure 5:**
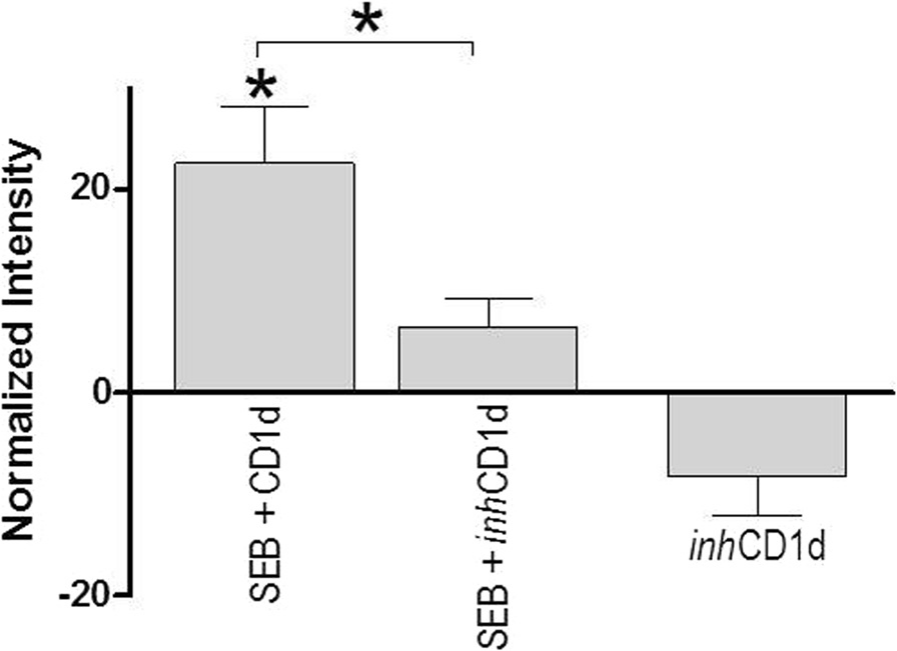
**Inhibition of CD1d impedes SEB binding to RPTECs: 1 × 10** ^**6**^ **cells/ml RPTECs were treated with mouse anti-CD1d antibody (1μl/ml) or PBS for 30 m; post-wash, the cells were exposed to 100 μg/ml SEB for 1 h.** Anti-SEB rabbit antibody followed by goat anti-rabbit FITC-conjugated secondary antibody was used for reporting purposes. A “no cell” control was used as baseline and the average was used to normalize intensity (Treated – Control ± SE) (*N* =5); Welch’s *t*-test *p* value cut-offs were designated by * < 0.05. The first bar (designated as SEB + CD1d) shows SEB-CD1d binding in the absence of the inhibitory anti-CD1d antibody. A significant reduction of signal was observed in presence of the inhibitory anti-CD1d antibody (second bar; designated as SEB + *inh*CD1d). Third bar plot (*inh*CD1d) shows the specificity of the anti-SEB primary antibody and the corresponding secondary antibody towards the anti-CD1d antibody in the absence of SEB exposure.

RPTECs were harvested as per the protocol described earlier, and 1 × 10^6^ cells/ml loaded in chamber slides were treated with 1 μg/ml rabbit anti-CD1d antibody (or PBS as negative control), termed as SEB + CD1d in Figure 5, for 30 m. Post-wash, the cells were exposed to 100 μg/ml SEB for 1 h. There was one additional set of controls termed as *inh*CD1d (Figure 5) that did not undergo the SEB treatment, but the rest of the protocol was carried out as previously described. Post-treatment, the cells were incubated on ice with anti-SEB rabbit antibody for 30 m on ice. The cells were washed twice, and the goat anti-rabbit FITC-conjugated secondary antibody suspended in 1× PBS was added and incubated for 5 m. Subsequently, the cells were washed, and resuspended in 1 ml of formaldehyde and PBS mix (1:1). After incubating at room temperature for 15 m, it was washed with 1× PBS and resuspended again in 1× PBS as before. The fluorescence intensity was estimated using the FX scanner (BioRad). A “no cell” control was used as the baseline, and their average read outs were used for normalization purpose. (Treated – Control ± SE) (*N* = 5). Welch’s corrected *t*-test *p* values * < 0.05 was used for all statistical notations.

## Results

### Binding efficiency and specificity of SEB to CD1d

The dotLab® System with diffractive optics technology (dot®) was used to systematically investigate the binding specificity of SEB to CD1d. In this study, biotinylated SEB (bt-SEB) was immobilized on a sensor equipped with avidin surface chemistry. The binding as the result of avidin-biotin interaction was displayed as an elevated upward ramp of DI at the end of around 500 s episode of BSA incubation (Figure 1A). Sequential BSA washing and the loading of CD1d:Ig to bt-SEB (each episode of incubation lasted for approximately 500 s) did not register the detectable increment of DI. The conjugation of SEB and CD1d took place nonetheless, as demonstrated by presenting an HRP-linked detector antibody (HRP-anti-mouse) that was conjugated to the Ig domain of CD1d:Ig duplex. Subsequently, DI was ramped as we introduced TMB, a precipitating substrate of HRP.

Four negative control assays were carried out to test the specificity of the conjugation between SEB and CD1d. In a mock surface control, nonspecific antibody bt-Dar was immobilized on the surface. CD1d:Ig followed by HRP-linked anti-mouse antibody was sequentially introduced as described earlier. TMB administration however, failed to increase the DI signals (Additional file 1: Figure S1A) denying the chance of nonspecific binding of CD1d in this assay. In an analyte blank control, BSA blank instead of CD1d:Ig was introduced on the immobilized SEB surface. Following the same assay sequence, weak signal amplification was recorded after TMB loading (Additional file 2: Figure S1B). Henceforth the specificity of the HRP-linked antibodies to CD1d:Ig was established.

Furthermore, we repeated this experiment, replacing CD1d:Ig with either (i) the Ig tag uncoupled from CD1d or (ii) HRP linked to nonspecific mouse antibody (HRP-1A11), which can only bind to human cardiac troponin T (Additional file 3: Figure S1C). Presentation of Ig-only substrate feebly increased DI while HRP-1A11 failed to increase DI after 600 s incubation.

The assay was further repeated with serially increasing concentrations of CD1d:Ig from 0.05 μg/ml to 1.0 μg/ml (Figure 1B), showing concurrent increment of the corresponding fluorescence signals.

### Longitudinal dynamics of the conjugation of SEB and CD1d on RPTECs’ surface

The longitudinal dynamics of RPTECs’ response to SEB exposures were evaluated by incubating the cells with SEB for increasing durations. To examine whether SEB treatment can affect the intracellular trafficking of CD1d, the distribution of CD1d expression on the RPTEC’s surface was monitored with and without SEB treatment. Compared to the untreated cells, the 1 h SEB treatment elevated CD1d expression on the RPTEC’s surface as reported by the Alexa 594-based fluorescence assay (Figure 2).

The outcome was further validated by the qPCR assay of the expression of CD1d after increased durations of SEB exposures. After 1 h SEB treatment, CD1d expression became significantly higher (*p* < 0.05) than that of the untreated control samples. Longer durations of SEB treatment retained the elevated level of CD1d mRNA transcriptomic expression (Figure 4).

The longitudinal dynamics of SEB binding to RPTEC were reported with accompanying CD1d expression on the cell surface (Figure 3). Sequential reporting of CD1d and SEB using antibodies with mutually exclusive binding epitopes showed co-localization of SEB and CD1d on the cell surface. After displaying a gradual increment until 1 h, the expression density gradually declined as reported after 2 h SEB exposure (Figure 3).

The reduction of SEB concentration conjugated to CD1d after 2 h incubation was verified with an immunoblotting study (Figure S2). Here, post-1 h and −2 h SEB exposure, the CD1d protein was surface-captured and SEB levels were reported by sequential addition of antibodies with mutually exclusive capturing epitopes. The concentration of SEB conjugated to CD1d was found diminished after 2 h SEB exposure.

### Consequences of inhibiting CD1d of RPTECs before SEB exposure

RPTECs were pre-treated with polyclonal anti-CD1d antibody before regular SEB treatment. The SEB reporting profile demonstrated the consequences of inhibiting CD1d from conjugating with the antigens. In Figure 5, the first bar from the left (SEB + CD1d) displayed the intensity of SEB conjugated to CD1d in the absence of the anti-CD1d antibody. A significant reduction of signal was observed in presence of the anti-CD1d antibody (second bar plot; SEB + *inh*CD1d) that inhibited the CD1d ability to conjugate SEB. A faint signal was nevertheless recorded. The third bar showed a negative signal (*inh*CD1d), which potentially attested that the anti-SEB primary antibody (and the corresponding secondary antibody) shared no common epitope with anti-CD1d polyclonal antibody.

## Discussion

Multiple studies reported the critical protective role played by the kidney in excretion of SEB [20-26]. RPTECs selectively express CD1d, which play a significant (*and* MHC II-independent) role in antigen sensing and recognition process [13,29-31]. The present study sought to elucidate the renal pathogenesis of SEB with the primary focus on the toxin’s interaction with CD1d expressed on RPTECs.

Time sensitive co-localization of SEB with CD1d expressed on RPTECs’ surface (Figure 3) was found in accordance with others’ findings [12,32,33,55]. The renal localization of SEB began as the toxin conjugated to CD1d; in fact, the co-localization, which began merely 15 m post-exposure possibly explained the etiology behind the ‘jumpstart’ of host immunity after CD1d-NKT cell conjugation [35].

We also showed that SEB conjugated directly to CD1d using the dot® platform (Figure 1A), a technology enabling us to probe the protein-ligand interaction in a real-time format with proven success at per the other industry standards such as BIAcore [47,48]. The direct and specific binding of renal CD1d with SEB was further attested by the selective immunoinhibition of CD1d (Figure 5) that inhibited SEB to bind RPTECs at all. This information is rather important in context to the evidence that showed no direct involvement of CD1d in tuberculosis antigen presentation [55,56] and to other reports suggesting non-conventional sAg presenting sites [18,19].

We paid particular attention to minimize the risk of false positive result of dot® assay by conducting an array of validation experiments. These included the probing of (i) the blank capturing agents on the surface that attested the specificity of the SEB and CD1d binding (Additional file 1: Figure S1A). (ii) The analyte blank control for CD1d attested the specificity of the HRP-linked antibody to CD1d:Ig (Additional file 2: Figure S1B). (iii) The analyte quantity control investigated the dose titration of the CD1d amount and verified the stoichiometric dynamics between CD1d and SEB (Figure 1B). (iv)The analyte tag negative control replacing the Ig tag control of CD1d with msIgG reconfirmed the specificity of Ig to HRP-like antibody (Additional file 3: Figure S1C). Finally (v) the HRP-linked detector antibody was replaced by HRP-1A11 as a nonspecific detector for CD1d that revalidated the same (Additional file 3: Figure S1C). Together these assay results imparted maximum confidence on the present inference about the direct and specific conjugation of SEB to CD1d.

Present methodology, however was not able to empirically measure the affinity between SEB and CD1d. This could be due to a weak SEB-CD1d interaction, which was potentially unlikely in light of the past studies that demonstrated high affinity of CD1d for glycolipids [57]; in fact, our data displaying a short duration required to form SEB-CD1d conjugates further supports this report [57]. As a more probable alternative, the requirement of a signal enhancing addendum (TMB, in this case) could be simply due to the fact that the reagents and the experimental conditions used *in vitro* in dot® experiments were different from what happens in the cell. A possible role of a secondary adjunct facilitating SEB CD1d conjugation in the cellular environment could not be ruled out; a more comprehensive study is required.

The fluorescence images obtained from RPTECs displayed the density of SEB conjugated with CD1d without the additional help from signal amplification agents. The result indicated that the SEB exposure prompted the elevation of CD1d expression on the cell surface (Figure 2).

As shown in Figure 3, the co-localization profile of SEB and CD1d displayed a gradual increment of their densities up to 1 h and concluded with a subsequent decline, which suggested the eventual trafficking of SEB. Also, Figure 2 suggested that the SEB challenges prompted CD1d expression on the cell surface after 1 h incubation, while the immunoblotting result (Additional file 4: Figure S2) validated the potential excretion of SEB. The enrichment of the cell-bound SEB depleted as the incubation period was extended to second hour. The present study was limited by not tracking the unbound SEB during the second hour of exposure.

Interestingly, the transcriptomic expression of CD1d remained elevated (Figure 4), possibly long after the SEB was sequestered. Of note, a previous study demonstrated a long half-life of CD1d [58]. These outcomes, coupled with Figure 2 displaying the SEB-induced the cell surface expression of CD1d may indicate that SEB regulates CD1d expression on RPTEC’s surface. Further investigation of CD1d in RPTECs could have major clinical interest, particularly in the context of understanding a robust system that enables facilitation of a delayed antigenic cleansing program [59].

The essential role of renal CD1d in SEB-associated pathogenesis was further validated with a fluorescence-based assay demonstrating the sequential inhibition of CD1d and preventing its binding to SEB (Figure 5). In agreement, multiple studies observed diminished host defense as a result of the targeted CD1d deficiency [37,40,59,60]. It was, however, beyond the scope of the present study to suggest putative SEB-induced toxemia through CD1d-independent routes. Such possibilities, nonetheless, have been suggested by the recruitment of CD1d-restricted T-cells [40,41] and invariant Natural Killer cells [15].

## Conclusion

In conclusion, the present study examined the renal pathogenesis of SEB, which revealed a potential functional role for CD1d in antigen recognition, cleansing and sequestration [12,22,26,27]. The binding specificity of CD1d and SEB was established. A rapid conjugation of CD1d with SEB implicated a possible high affinity between the two molecules, particularly in RPTECs. Such prompt interaction may facilitate the rapid surge of cytokines triggered by the crosstalk between CD1d and NKT cells [35].

The display of the co-localization of SEB and CD1d at the RPTEC’s surface and the decline of the SEB population on the cell surface after CD1d immunoinhibition suggested that RPTECs undertake an exclusive CD1d-mediated antigen presentation process. The transcriptomic elevation (and cell surface expression) of CD1d in RPTECs was concurrent with SEB exposure, which remained elevated potentially long after SEB trafficking. Together, a feedback-controlled conjugation of SEB and CD1d is suggested in carrying out the recognition, cleansing and sequestration of SEB. Further investigation is required to establish this CD1d-mediated mechanism as a potential downstream mechanism of SEB excretion and a prospective therapeutic target.

## Additional files

Additional file 1: Figure S1A. Validation of the kinetics of SEB-CD1d interaction: Mock surface control: nonspecific surface antibody (bt-Dar) did not bind to CD1d: As a mock surface control for bt-SEB, nonspecific antibody bt-Dar (identified by the dashed circle) was immobilized on the surface. TMB loading failed to increase the DI signals, indicating that there wasn’t nonspecific binding of CD1d to bt-Dar. Additional file 2: Figure S1B. Validation of the kinetics of SEB-CD1d interaction: Analyte control, BSA blank replacement of CD1d failed to emit signal: No CD1d:Ig fusion protein was presented in the assay, instead, a blank BSA wash was performed. The changed reagent is identified by the dashed circle. The specificity of horse anti-mouse HRP-conjugated antibody to CD1d:Ig fusion protein was reported with no increment of DI trace after TMB presentation. Additional file 3: Figure S1C. Validation of the kinetics of SEB-CD1d interaction: Analyte tag control: Signal decreased as (a) the Ig tag uncoupled from CD1d was presented: The normalized DI signal during 600 s after the introduction of TMB was plotted against the concentrations of CD1d from 1.0 μg/ml as reported in Additional file 1: Figure S1A (red line). A polyclonal anti-mouse antibody (msIgG, a mixture containing all subclass of IgG including IgG1) was presented as an analyte-negative. The rest of the assay was same as described in Additional file 1: Figure S1A. The Ig tag without CD1d failed to signal as reported in the blue line (msIgG (a)). The signal decreased as (b) HRP conjugated with nonspecific antibody was presented: HRP-1A11, a HRP-linked nonspecific mouse antibody (only capable of binding human cardiac troponin T), was employed as a HRP detector negative control. The rest of the assay was the same as described in Additional file 1: Figure S1A. The nonspecific mouse antibody failed to couple with CD1d:Ig fused protein, reporting no signal as indicated in the green line (HRP-1A11 (b)). Additional file 4: Figure S2. Time course study of SEB binding to RPTECs: Immunoblotting to assess SEB levels by measuring bound CD1d: 1 x 10^6^ cells/ ml RPTECs were exposed to 100 μg/ml SEB for two time durations, namely 1 h and 2 h; controls were not exposed to SEB. Cells were coupled with mouse anti-CD1d antibody and immunoprecipitated with protein G magnetic beads. Immunoblotting was completed by presenting the conjugate to anti-SEB rabbit antibody followed by goat anti-rabbit HRP conjugated secondary antibody.
