# Supplementary figures and images for: Characterization of the interaction of staphylococcal enterotoxin B with CD1d expressed in human renal proximal tubule epithelial cells
Source: BMC Microbiol. 2015 Feb 4;15(1):12. doi: 10.1186/s12866-015-0344-5 (PMC4327782; doi:10.1186/s12866-015-0344-5)

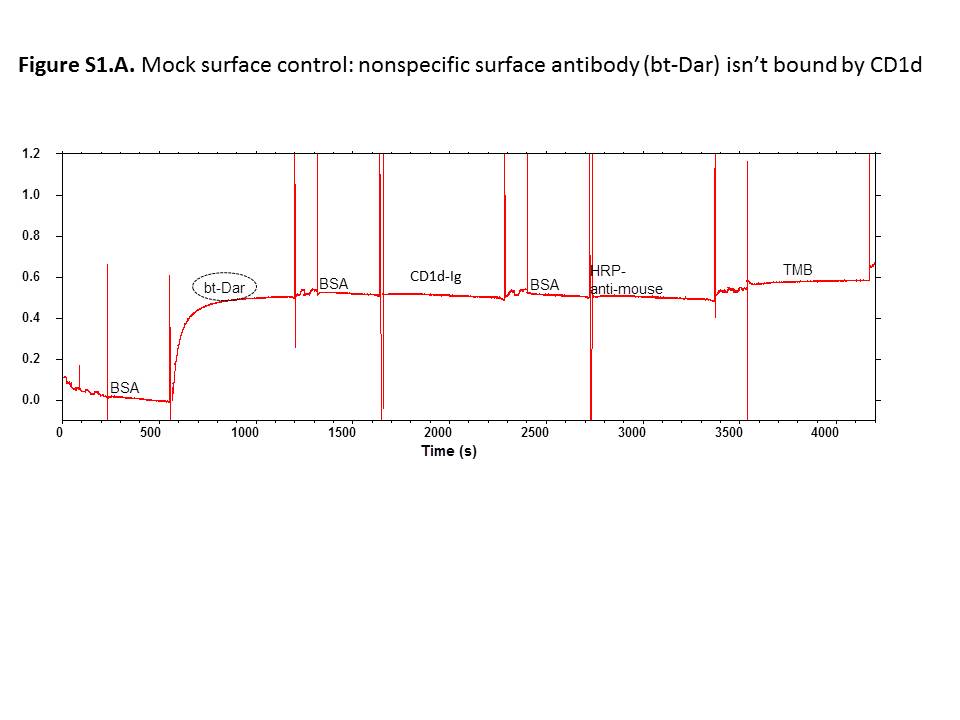

Supplement: Additional file 1: Figure S1A. — Validation of the kinetics of SEB-CD1d interaction: Mock surface control: nonspecific surface antibody (bt-Dar) did not bind to CD1d: As a mock surface control for bt-SEB, nonspecific antibody bt-Dar (identified by the dashed circle) was immobilized on the surface. TMB loading failed to increase the DI signals, indicating that there wasn’t nonspecific binding of CD1d to bt-Dar. [file 12866_2015_344_MOESM1_ESM.jpeg]

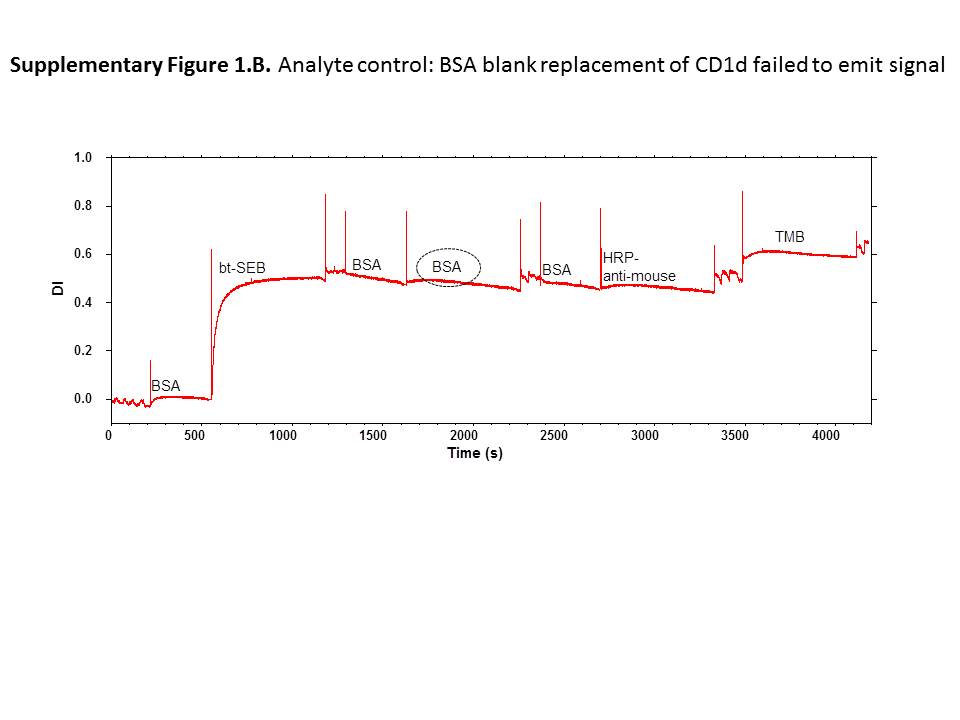

Supplement: Additional file 2: Figure S1B. — Validation of the kinetics of SEB-CD1d interaction: Analyte control, BSA blank replacement of CD1d failed to emit signal: No CD1d:Ig fusion protein was presented in the assay, instead, a blank BSA wash was performed. The changed reagent is identified by the dashed circle. The specificity of horse anti-mouse HRP-conjugated antibody to CD1d:Ig fusion protein was reported with no increment of DI trace after TMB presentation. [file 12866_2015_344_MOESM2_ESM.jpeg]

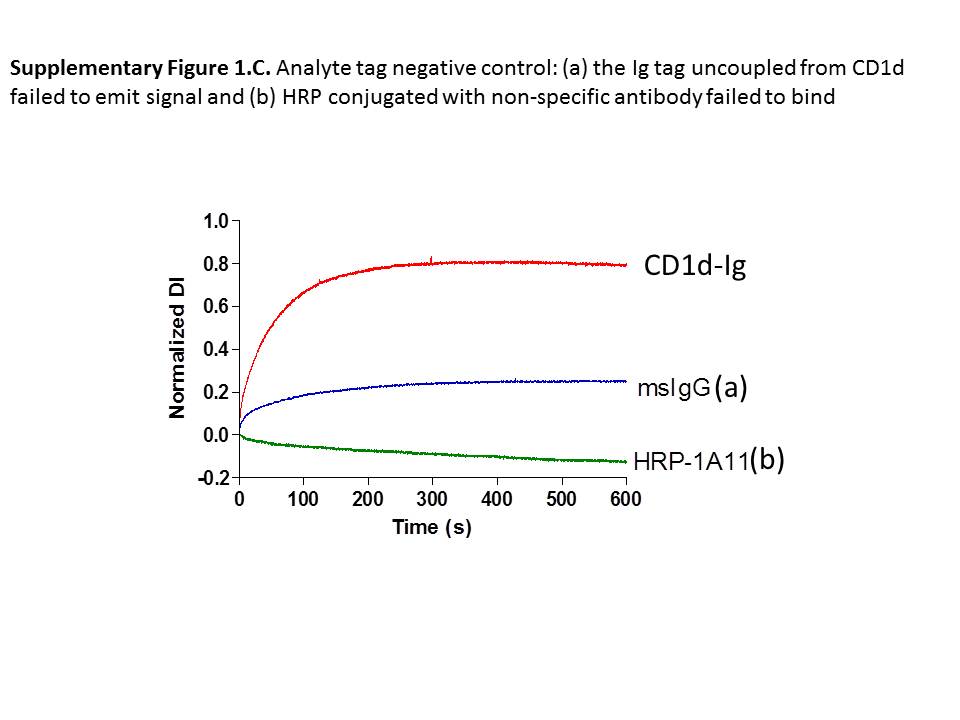

Supplement: Additional file 3: Figure S1C. — Validation of the kinetics of SEB-CD1d interaction: Analyte tag control: Signal decreased as (a) the Ig tag uncoupled from CD1d was presented: The normalized DI signal during 600 s after the introduction of TMB was plotted against the concentrations of CD1d from 1.0 μg/ml as reported in Additional file 1: Figure S1A (red line). A polyclonal anti-mouse antibody (msIgG, a mixture containing all subclass of IgG including IgG1) was presented as an analyte-negative. The rest of the assay was same as described in Additional file 1: Figure S1A. The Ig tag without CD1d failed to signal as reported in the blue line (msIgG (a)). The signal decreased as (b) HRP conjugated with nonspecific antibody was presented: HRP-1A11, a HRP-linked nonspecific mouse antibody (only capable of binding human cardiac troponin T), was employed as a HRP detector negative control. The rest of the assay was the same as described in Additional file 1: Figure S1A. The nonspecific mouse antibody failed to couple with CD1d:Ig fused protein, reporting no signal as indicated in the green line (HRP-1A11 (b)). [file 12866_2015_344_MOESM3_ESM.jpeg]

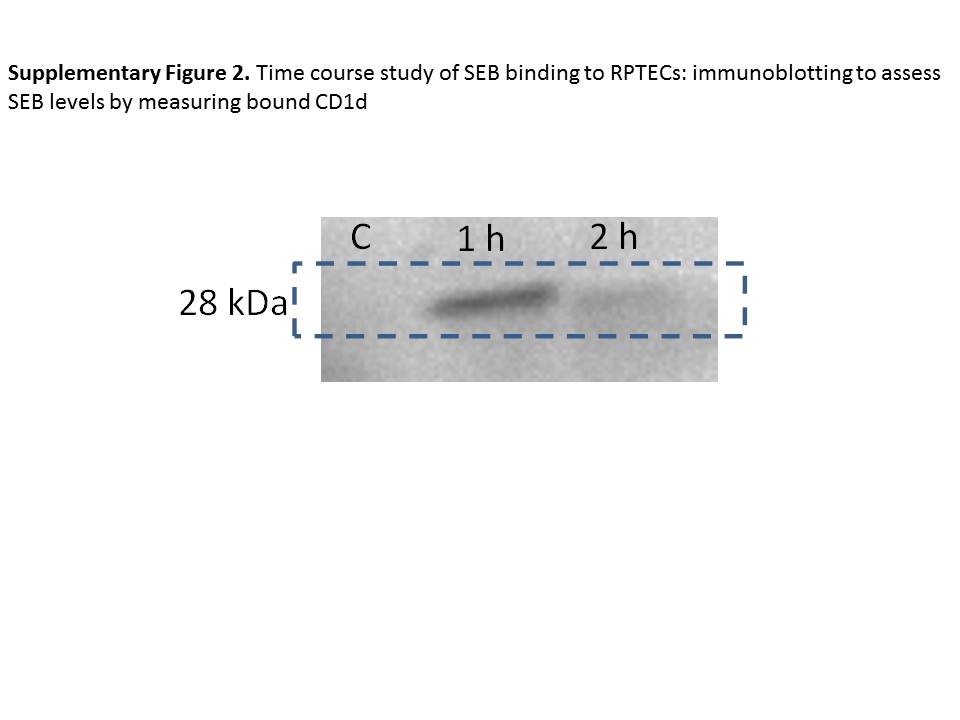

Supplement: Additional file 4: Figure S2. — Time course study of SEB binding to RPTECs: Immunoblotting to assess SEB levels by measuring bound CD1d: 1 x 10^6^ cells/ ml RPTECs were exposed to 100 μg/ml SEB for two time durations, namely 1 h and 2 h; controls were not exposed to SEB. Cells were coupled with mouse anti-CD1d antibody and immunoprecipitated with protein G magnetic beads. Immunoblotting was completed by presenting the conjugate to anti-SEB rabbit antibody followed by goat anti-rabbit HRP conjugated secondary antibody. [file 12866_2015_344_MOESM4_ESM.jpeg]
